# Supplementary material for: Genomic and epigenomic integrative subtypes of renal cell carcinoma in a Japanese cohort
Source: Nat Commun. 2023 Dec 16;14:8383. doi: 10.1038/s41467-023-44159-1 (PMC10725467; doi:10.1038/s41467-023-44159-1)
Supplement: Supplementary file 1 — Supplementary Information [file 41467_2023_44159_MOESM1_ESM.pdf]

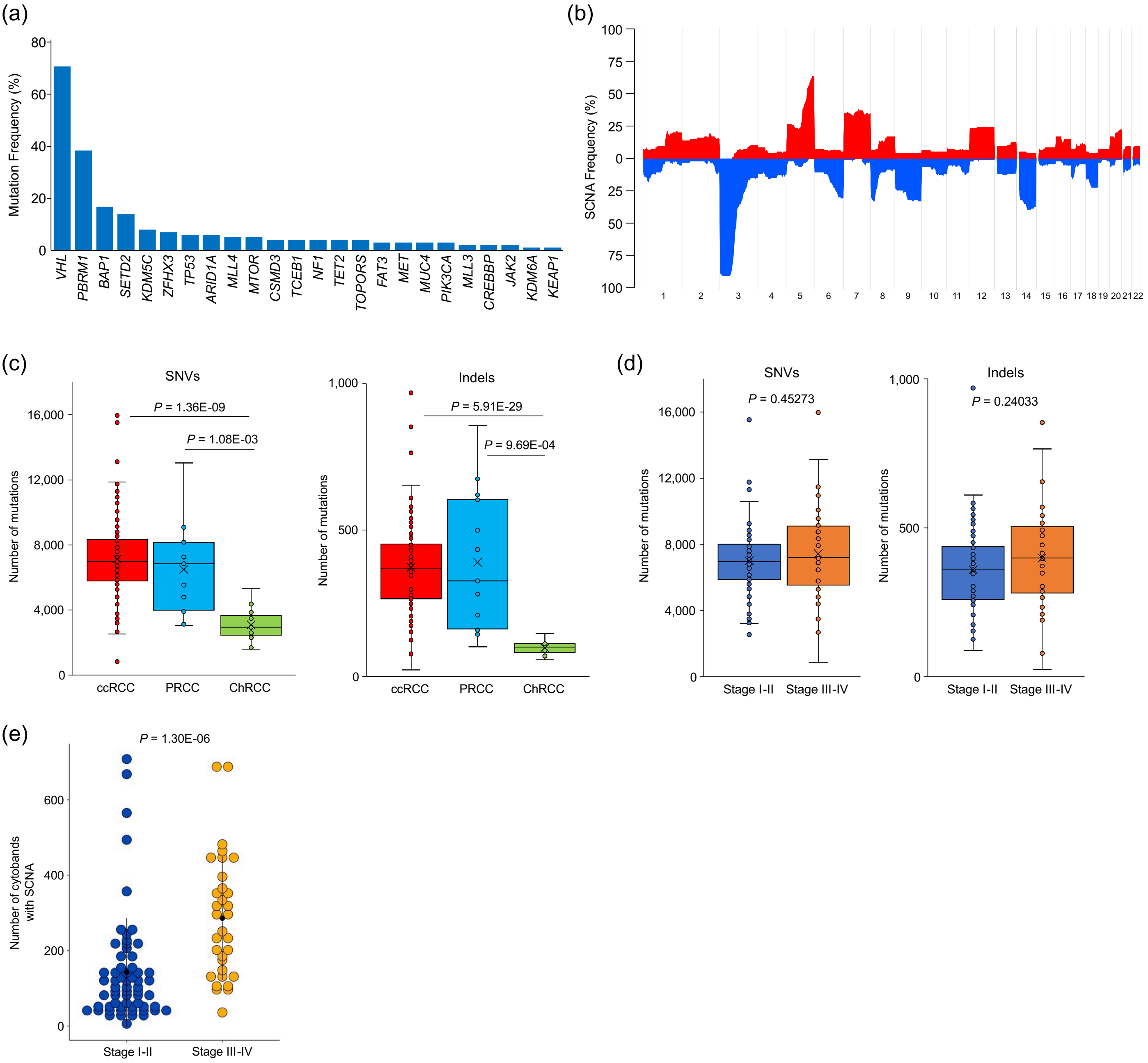

Supplementary Figure 1. Genomic alteration of RCCs

(a) Frequency of nonsynonymous mutations in ccRCC cases. (b) Frequency of ccRCC cases with SCNAs. Somatic copy number gains and deletions are indicated in red and blue, respectively. (c) The numbers of SNVs and short indels in ccRCC, PRCC, and ChRCC. ccRCC n=102, PRCC n=13, ChRCC n=11. (d) The numbers of the SNVs and indels in Stage I-II (n=65) and Stage III-IV cases (n=37) , respectively. (e) The number of cytobands with SCNAs in advanced-stage (n=32) and early-stage cases (n=62). Dots and whiskers represent the median and the ± 1.5 × the IQR. *P*-values were calculated using the two-sided Wilcoxon rank-sum test. (c), (d) Box plots show the median (lines), IQR (boxes), and ± 1.5 × the IQR (whiskers). *P*-values were calculated using the two-sided Wilcoxon rank-sum test.

Source data are provided as Source Data file.

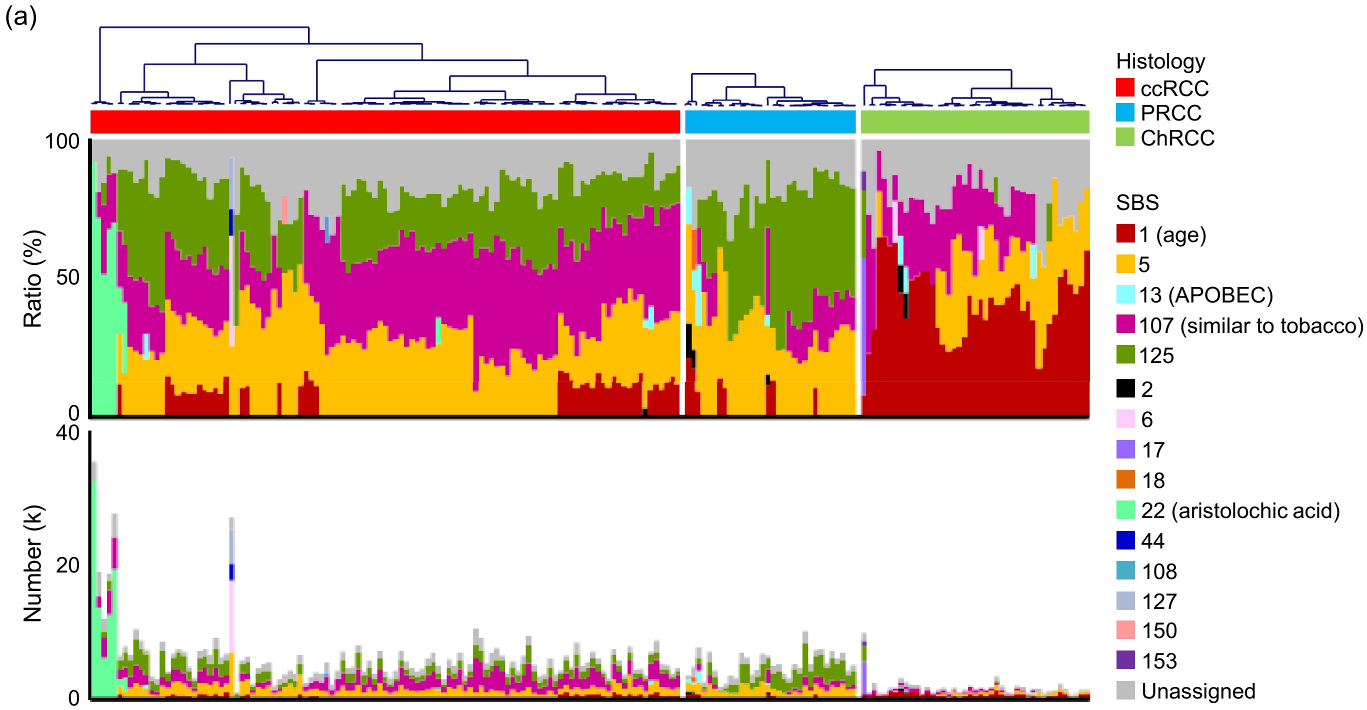

Supplementary Figure 2. Substitution mutational signatures in PCAWG RCC cases

(a) Signal-mutational signatures of 186 PCAWG RCC cases (111 ccRCC, 32 PRCC, 43 ChRCC). The bar represents histological types. The upper and lower histograms show the relative and absolute values of the mutational signatures, respectively.

Source data are provided as Source Data file.

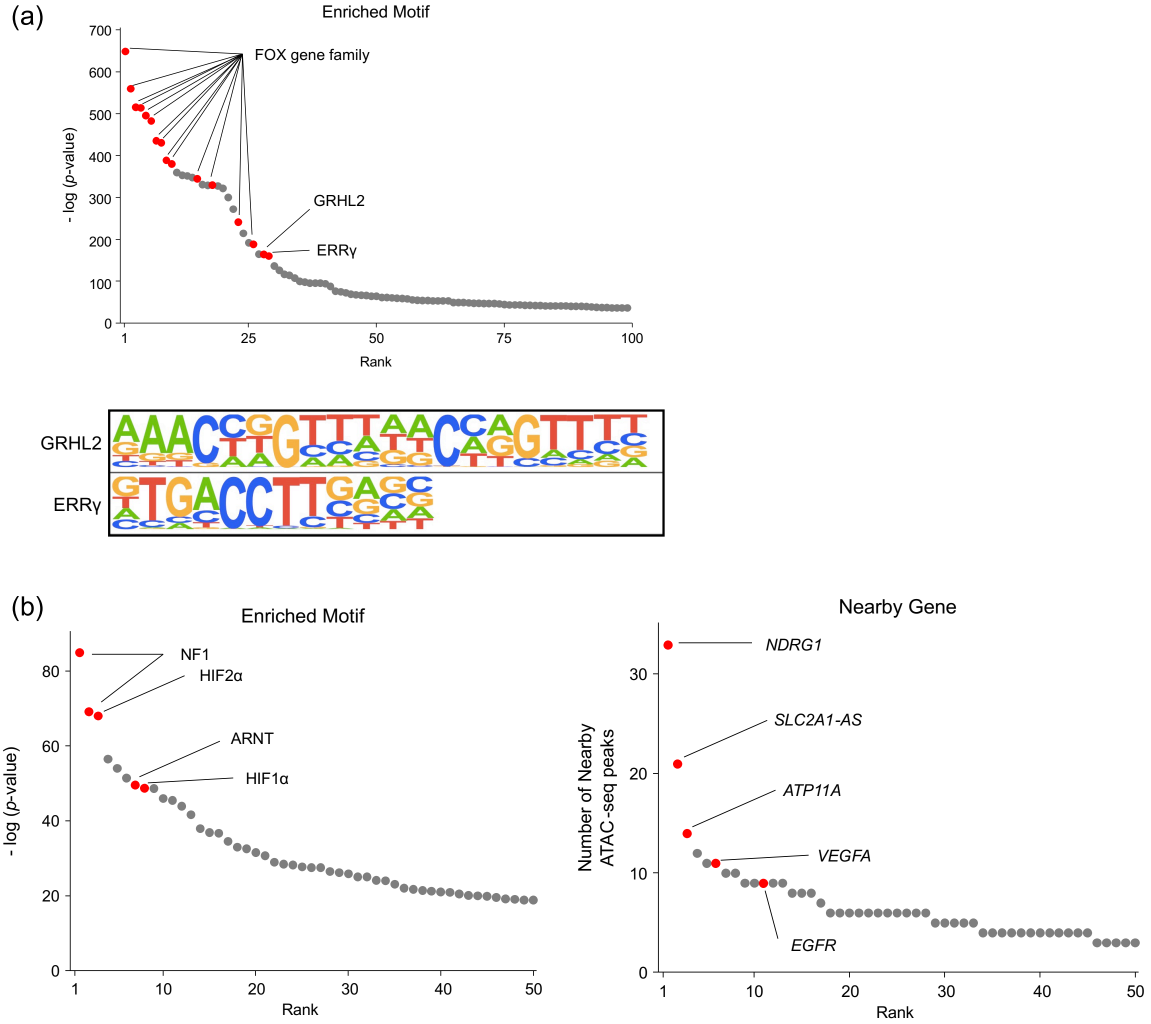

Supplementary Figure 3. Chromatic features of RCCs

(a) Enriched motifs in peak cluster 1 and sequence logos of GRHL2 and ERRγ. Each dot represents an individual motif sequence, and the dot plot represents one-sided *P*-values calculated by HOMER and rank. (b) Enriched motifs and nearby genes in peak cluster 3. The left dot plot represents one-sided *P*-values calculated by HOMER and rank. The right dot plot shows the number of peaks in the regions of genes.

Source data are provided as Source Data file.

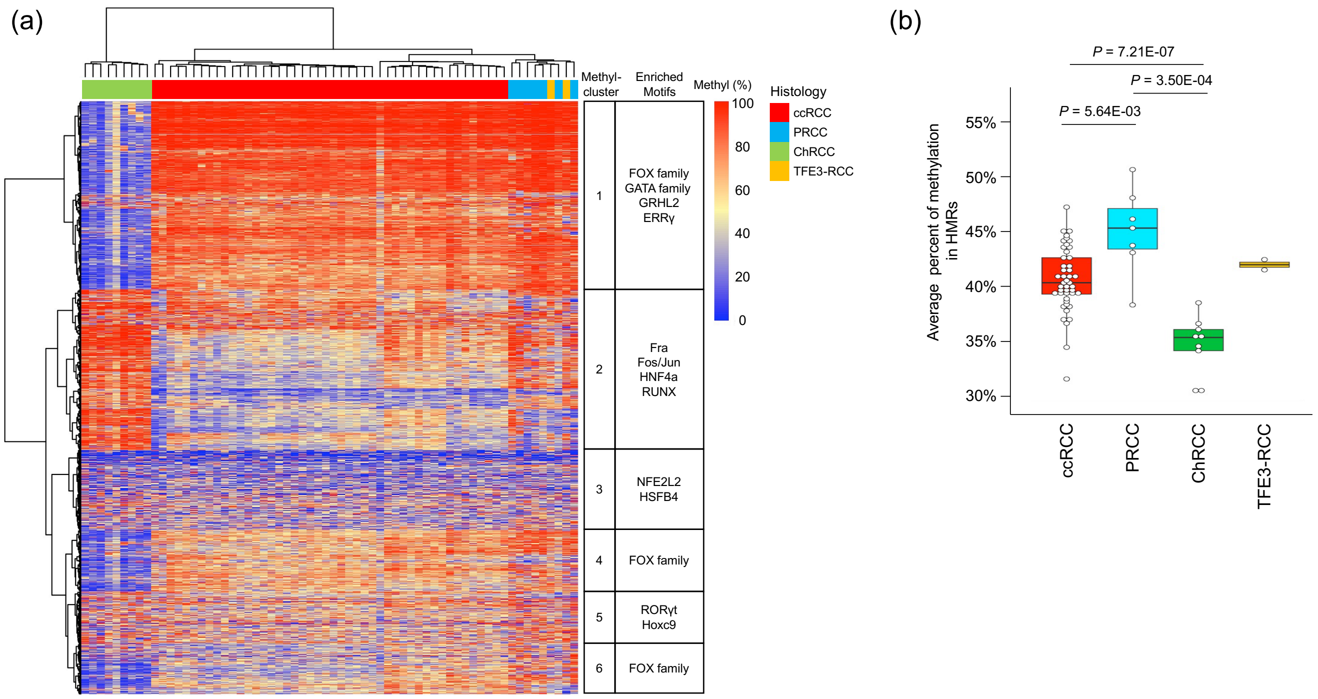

Supplementary Figure 4. Methylation profile of RCCs

(a) Heatmap of methylation status. Each row represents a region extracted from HMRs by high variance, and each column represents a case. Heatmap colours represent methylation percentages. Hierarchical clustering was performed using the ward.D2 method and Euclidean distance. One thousand HMRs are divided into six clusters, and enriched motifs in each cluster are shown to the right. (b) The average methylation percentage in HMRs. Box plots show the median (lines), IQR (boxes), and $\pm$ 1.5 $\times$ the IQR (whiskers), and individual samples (dots). *P*-values were calculated using the two-sided Wilcoxon rank-sum test. ccRCC n=46, PRCC n=7, ChRCC n=9, TFE3-RCC n=2.

Source data are provided as Source Data file.

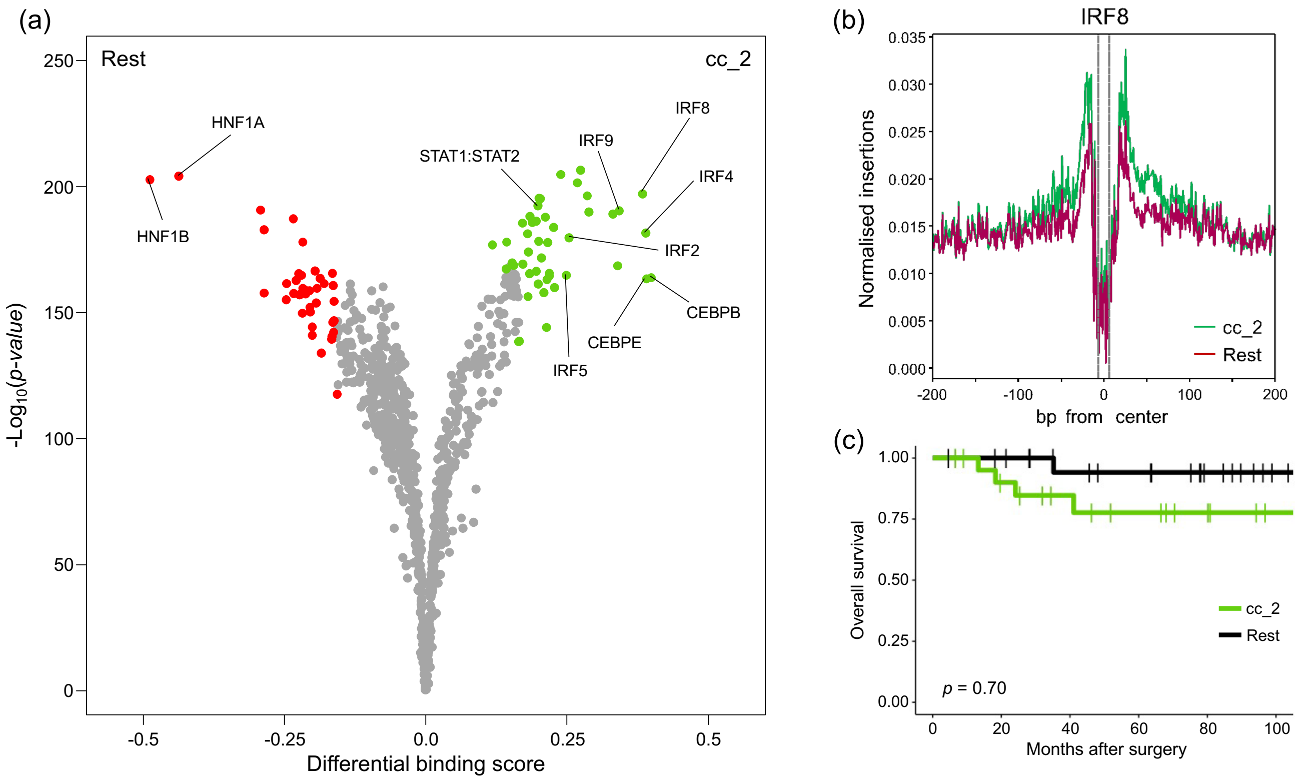

Supplementary Figure 5. Footprint analysis of cc_2

(a) Differential binding scores comparing cc_2 to the other two epi-subtypes (cc_1 and cc_3). Each dot represents an individual motif sequence, and green and red dots indicate the predominant sequences in cc_2 and the other two epi-subtypes, respectively. One-sided *P*-values were calculated by TOBIAS. (b) TF footprinting of the IRF8 motif in cc_2 and the other two epi-subtypes, shown in green and red, respectively. The regions between the dotted lines show the motif centre of IRF8. (c) Kaplan-Meier curves show the overall survival of cc_2 and the other two epi-subtypes. The *P*-value was calculated using the log-rank test.

Source data are provided as Source Data file.

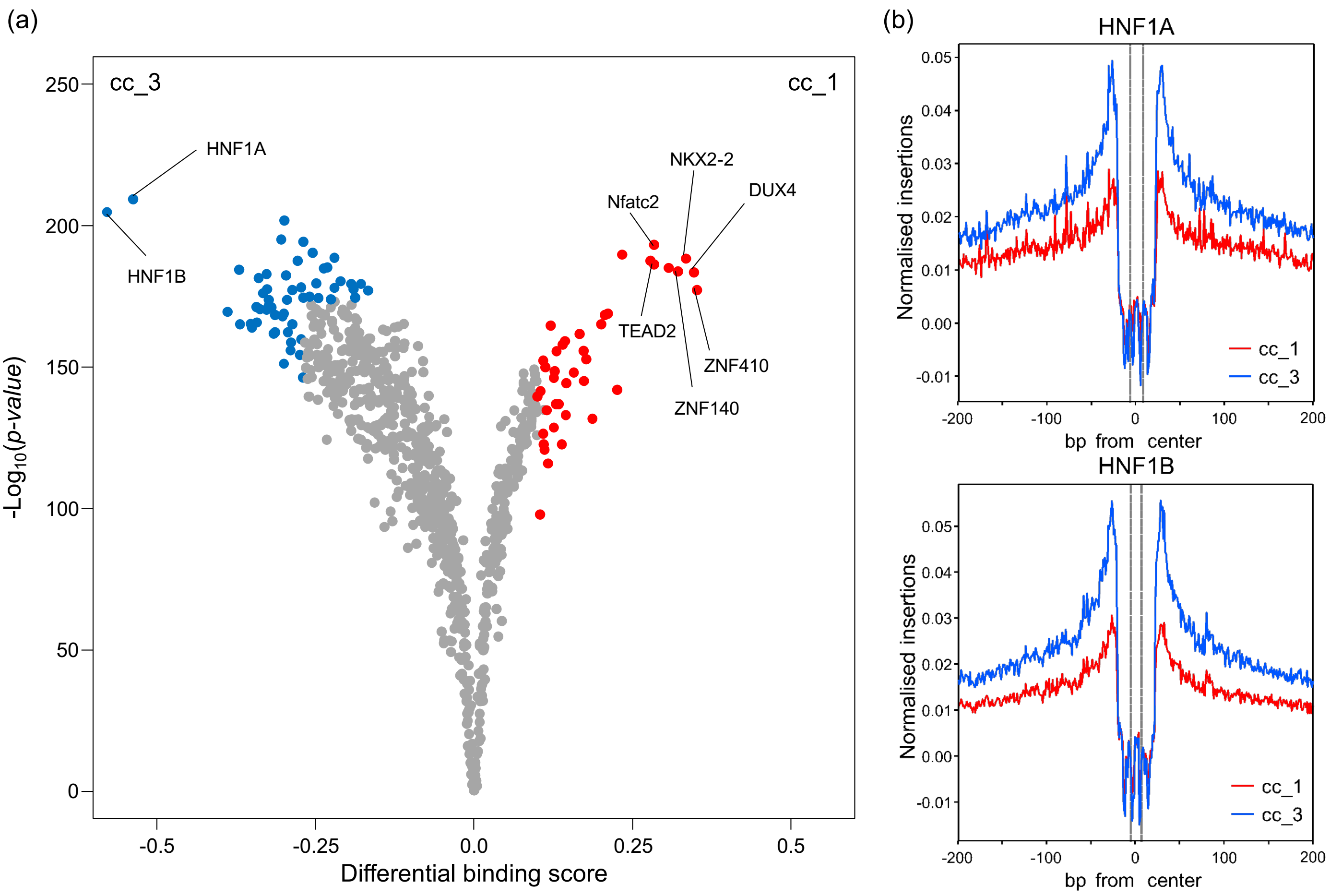

Supplementary Figure 6. Footprint analysis of cc_1 and cc_3

(a) Differential binding scores comparing cc_1 and cc_3. Each dot represents an individual motif sequence, and red and blue dots indicate motifs predominant in cc_1 and cc_3, respectively. One-sided *P*-values were calculated by TOBIAS. (b) TF footprint analysis of the HNF1A and HNF1B motifs in cc_1 and cc_3; red indicates cc_1, and blue indicates cc_3. The regions between the dotted lines show the motif centres of HNF1A and HNF1B.

Source data are provided as Source Data file.

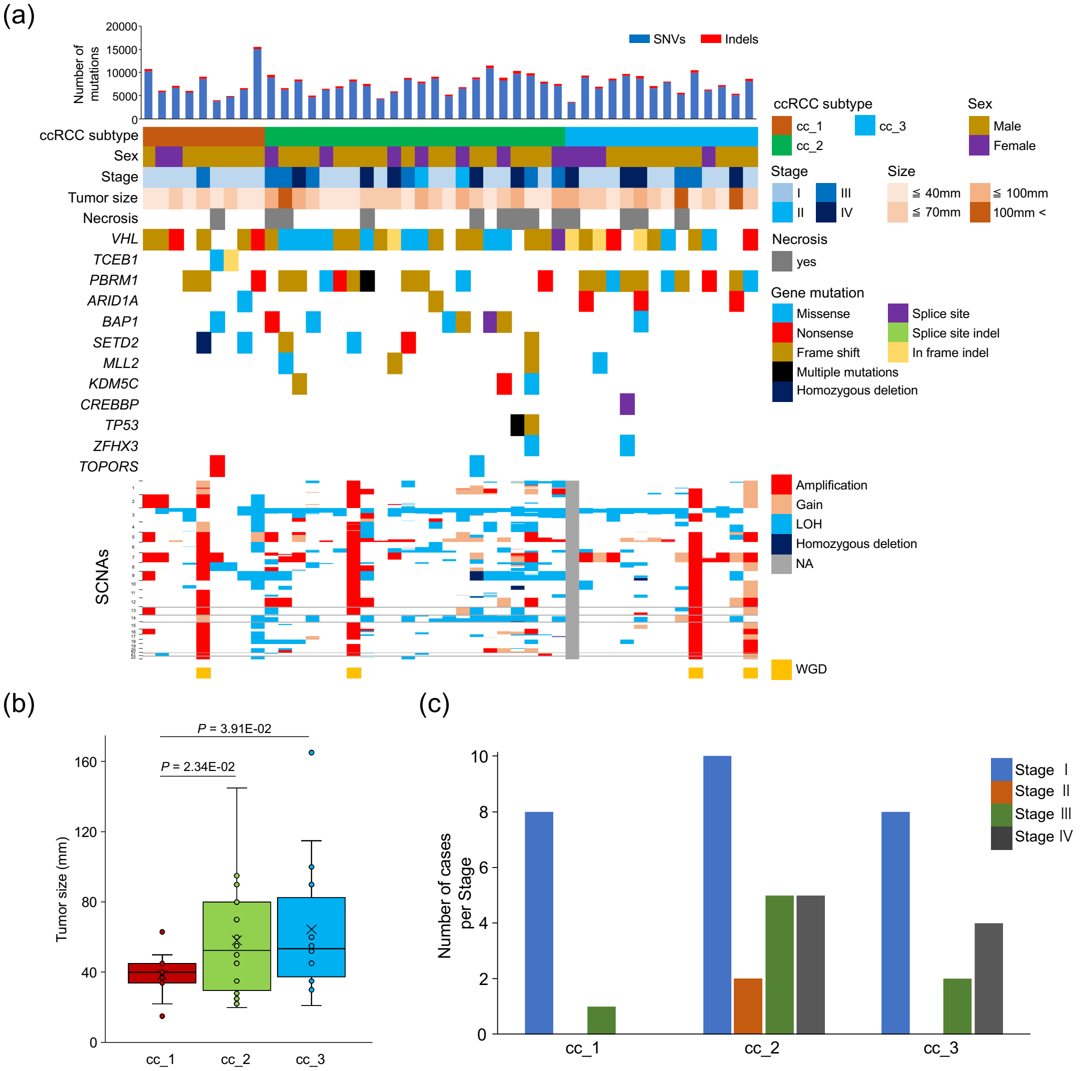

Supplementary Figure 7. Genomic and clinical profiles of ccRCC epi-subtypes

(a) Somatic alteration landscape of 45 ccRCC samples classified into cc_1-3. The top histogram represents the number of SNVs and short indels across the whole genome. The upper heatmap shows data on histology, sex, stage, tumour size, and necrosis. The middle heatmap shows frequent nonsynonymous mutations. The lower heatmap shows the SCNAs. N/A, not applicable. (b) Maximum tumour diameter at the time of surgery. Box plots show the median (lines), IQR (boxes), and $\pm$ 1.5 $\times$ the IQR (whiskers), and individual samples (dots). cc_1 n=9, cc_2 n=22, cc_3 n=14. *P*-values were calculated using the two-sided Welch's t-test. (c) Number of cases in each epi-subtype for each stage classification.

Source data are provided as Source Data file.

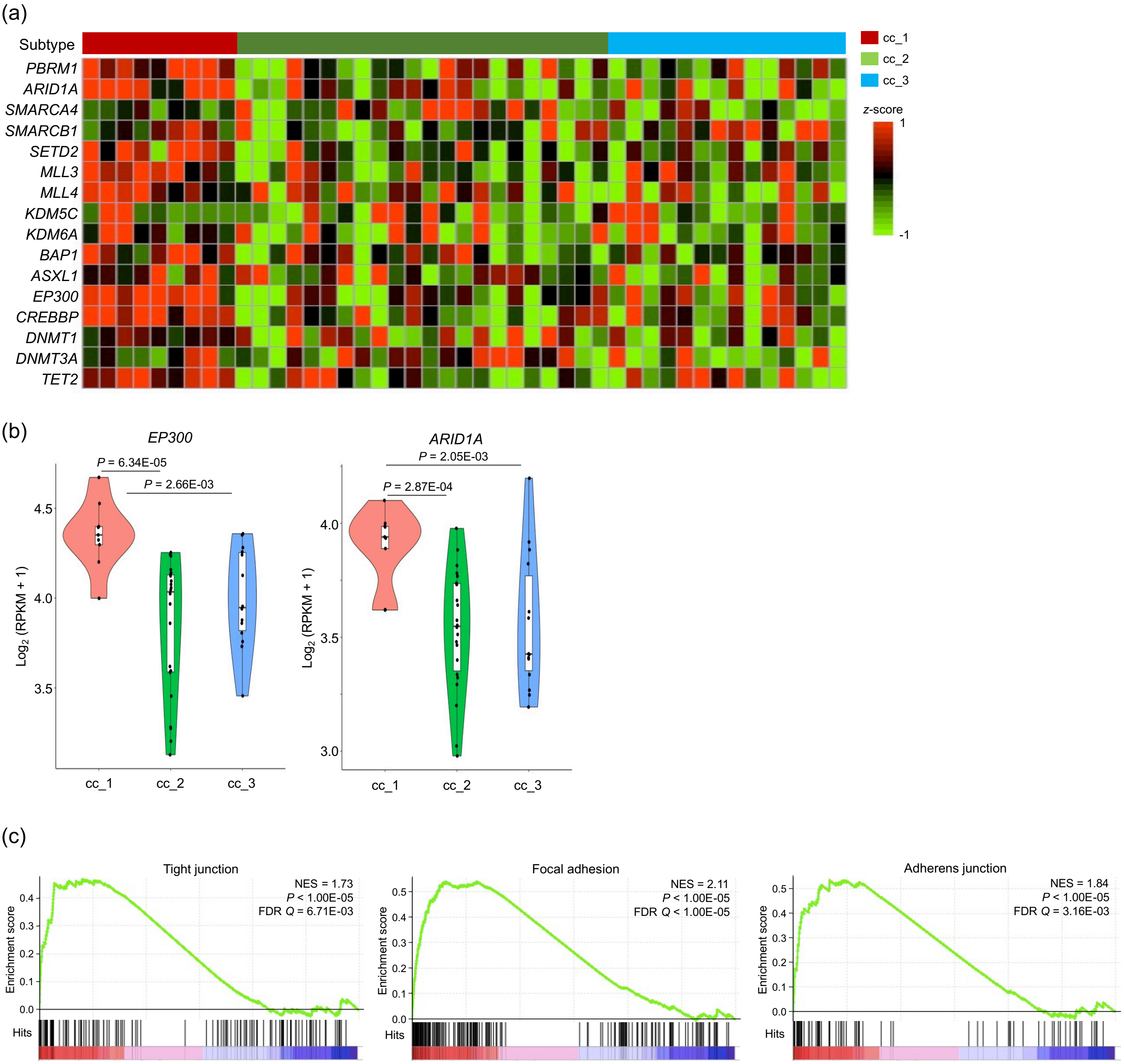

Supplementary Figure 8. Transcriptomic features of ccRCC epi-subtypes

(a) Relative expression of chromatin modifiers. The heatmap shows the *z*-scores of the gene expressions of chromatin modifiers. The top bar indicates ccRCC epi-subtypes. (b) The gene expressions of *EP300* and *ARID1A* in ccRCC epi-subtypes. Violin plots show the median (lines), IQR (boxes), and ± 1.5 × the IQR (whiskers), and individual samples (dots). *P*-values were calculated using the two-sided Wilcoxon rank-sum test. cc_1 n=9, cc_2 n=22, cc_3 n=14. (c) GSEA of tight junctions, adherens junctions, and focal adhesions registered in the KEGG pathway, comparing cc_1 to the other two epi-subtypes (cc_2 and cc_3).

Source data are provided as Source Data file.

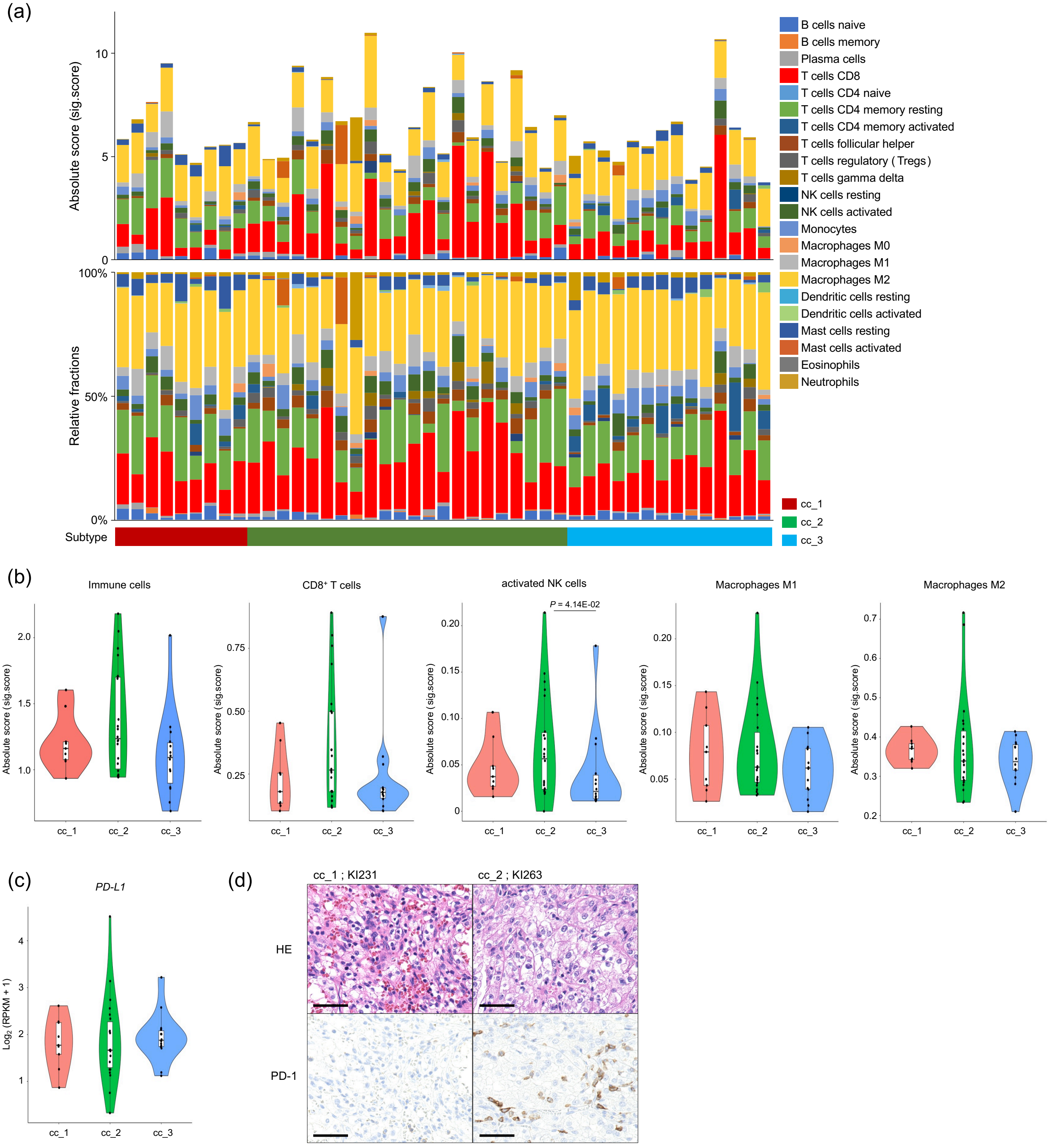

Supplementary Figure 9. Immune environment of ccRCC epi-subtypes

(a) CIRBERSORTx results. The upper and lower histograms show the absolute and relative scores of the immune fractions, respectively. The bottom bar indicates ccRCC epi-subtypes. (b) CIBERSORTx absolute scores of total immune cells and immune fractions. (c) Gene expressions of *PD-L1* in each epi-subtype. (d) HE staining and IHC of PD-1 in samples ID KI231 and KI263. Scale bars, 50µm. (b), (c) Violin plots show the median (lines), IQR (boxes), and ± 1.5 × the IQR (whiskers), and individual samples (dots). *P*-values were calculated using the two-sided Wilcoxon rank-sum test. cc_1 n=9, cc_2 n=22, cc_3 n=14.

Source data are provided as Source Data file.

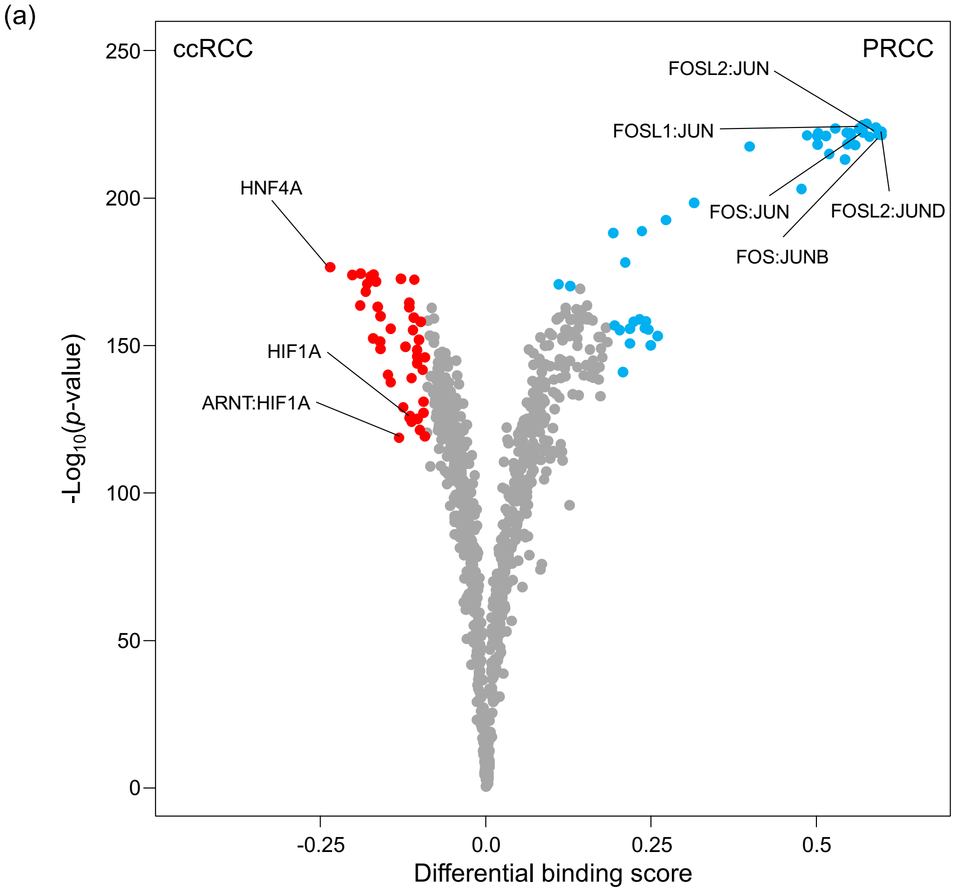

Supplementary Figure 10. Footprint analysis of PRCC and ccRCC

(a) Differential binding scores comparing PRCC to ccRCC. Each dot represents an individual motif sequence, and blue and red dots are predominant in PRCC and ccRCC, respectively. One-sided *P*-values were calculated by TOBIAS.

Source data are provided as Source Data file.

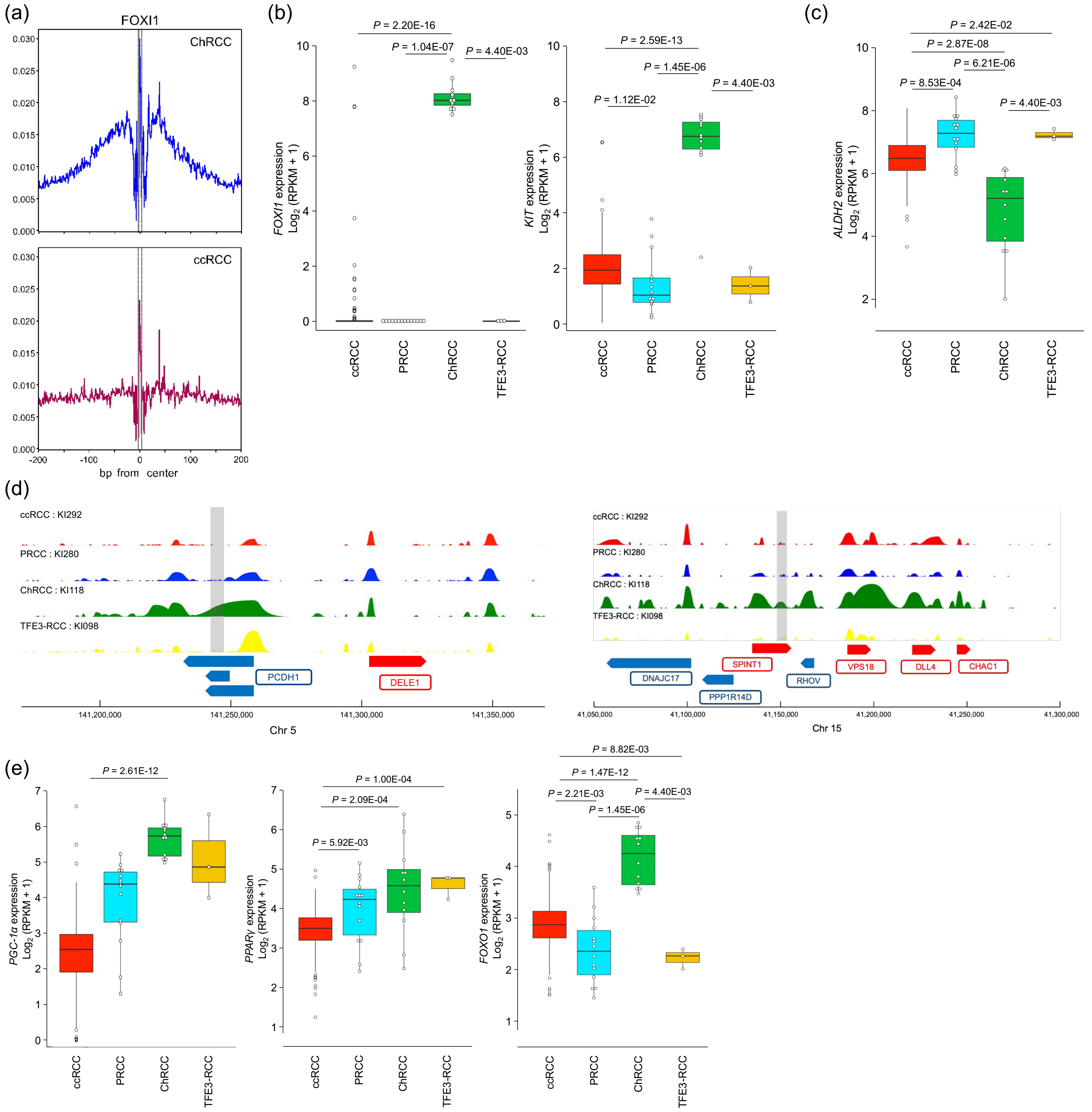

Supplementary Figure 11. Transcriptomic and epigenetic features of ChRCC

(a) TF footprint analysis of FOXI1 motifs in ChRCC and ccRCC. The regions between the dotted lines show the motif centre of FOXI1. (b) The gene expression of *FOXI1* and *KIT* in RCC. (c) The gene expression of *ALDH2* in RCC. (d) ATAC-seq peaks in the regions near *PCDH1* and *SPINT1*. The grey lines represent the predicted GRHL2 target regions [39]. Each three-digit number beginning with KI indicates a sample ID. (e) The gene expressions of *PGC-1α*, *PPARγ*, and *FOXO1*, co-activators of ERRγ, in RCC. (b), (c), (e) Box plots show the median (lines), IQR (boxes), and ± 1.5 × the IQR (whiskers) individual samples in PRCC, ChRCC, and TFE3-RCC (dots), and samples of the outliers in ccRCC (dots). *P*-values were calculated using the two-sided Wilcoxon rank-sum test. ccRCC n=258, PRCC n=14, ChRCC n=12, TFE3-RCC n=3.

Source data are provided as Source Data file.

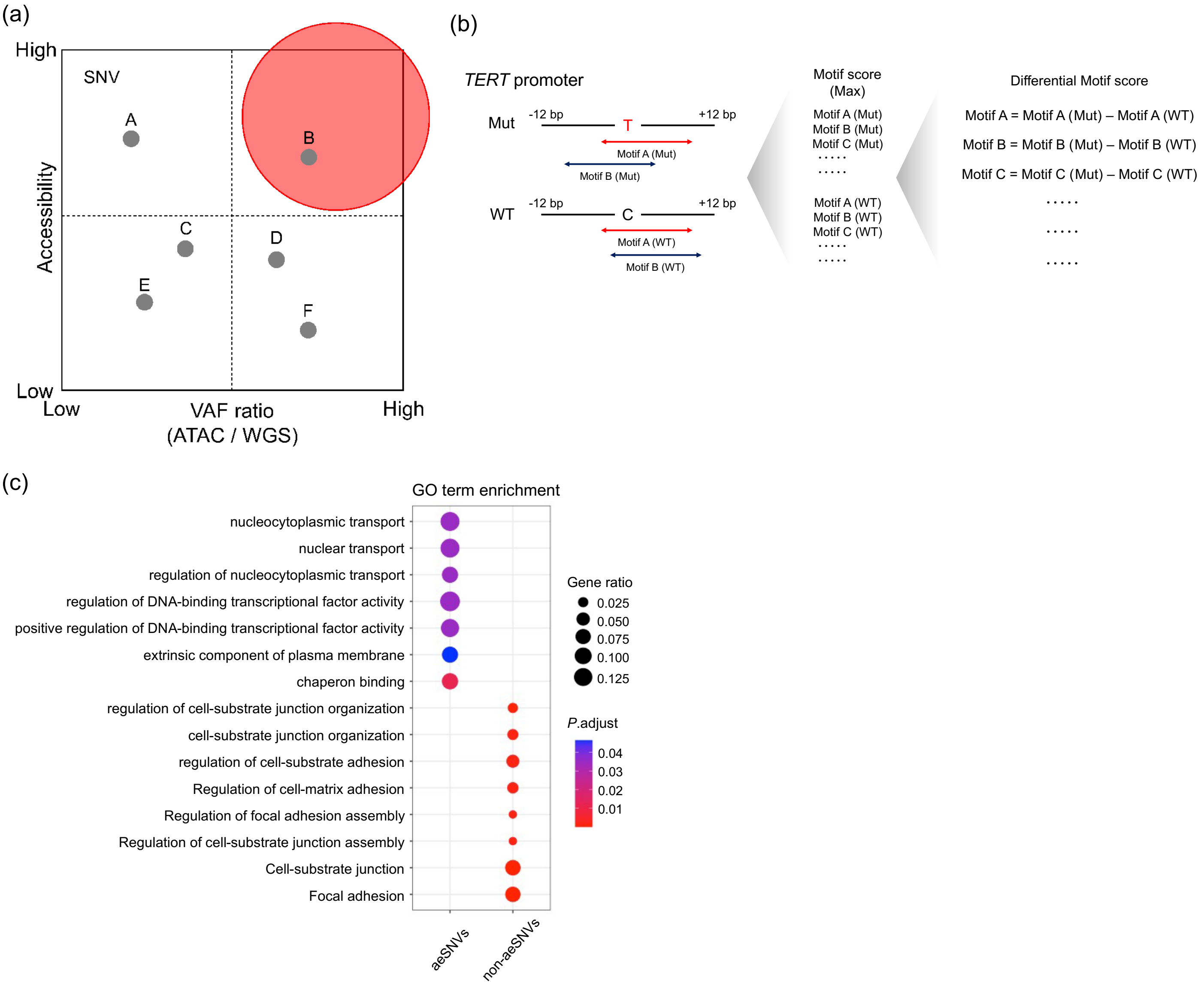

Supplementary Figure 12. Promoter SNVs in RCC

(a) The figure shows the VAF ratio (ATAC-seq VAF/WGS VAF) and accessibility on the X and Y axes, respectively. Each dot represents an individual mutation. SNVs in the upper right quadrant are expected to increase chromatin accessibility. (b) Mutant and wild-type strings were constructed ± 12 bp from the *TERT* mutation. Mut, mutation string; WT, wild-type string. We extracted the motif positions with the highest motif scores that the motifs overlap the SNV in WT and Mut, respectively. Finally, differential motif scores were computed. (c) GSEA of the genes with aeSNVs and non-aeSNVs using GO term.

Source data are provided as Source Data file.
