# Supplementary material for: Genomic and epigenomic integrative subtypes of renal cell carcinoma in a Japanese cohort
Source: Nat Commun. 2023 Dec 16;14:8383. doi: 10.1038/s41467-023-44159-1 (PMC10725467; doi:10.1038/s41467-023-44159-1)
Supplement: Supplementary file 3 — Description of Additional Supplementary Files [file 41467_2023_44159_MOESM3_ESM.pdf]

Description of Additional Supplementary Files

Supplementary Data 1. Clinical data of Japanese 287 RCC cases

Supplementary Data 2. Clinical data of RCC cases we performed WGS for in this study

Supplementary Data 3. Fusion genes in TFE3-translated RCC cases

Supplementary Data 4. Fusion genes identified in RCC

Supplementary Data 5. Focally amplified genes and the number of amplified cases

Supplementary Data 6. Homozygously deleted genes and the number of deleted cases in RCC

Supplementary Data 7. Nonsynonymous mutations in ccRCC

Supplementary Data 8. Nonsynonymous mutations in PRCC

Supplementary Data 9. Nonsynonymous mutations in ChRCC

Supplementary Data 10. Nonsynonymous mutations in TFE3-RCC

Supplementary Data 11. Significantly different mutated genes between early-stage and advanced-stage ccRCC cases

Supplementary Data 12. Significantly different mutated genes between ccRCC with necrosis and without it

Supplementary Data 13. P-values of the Figure 1c

Supplementary Data 14. Mutation signature analysis for PCAWG RCC's samples

Supplementary Data 15. TSS enrichment score of our ATAC-seq data

Supplementary Data 16. Enriched motifs in each peak cluster

Supplementary Data 17. Nearby gene in each peak cluster

Supplementary Data 18. Enriched motifs in each methyl-cluster

Supplementary Data 19. Multivariate analysis of ccRCC epi-subtypes

Supplementary Data 20. P-values of the Figure 4a

Supplementary Data 21. Differential motif scores on TERT mutation

Supplementary Data 22. Promoter mutation we analysed in Figure 7

Supplementary Data 23. Variant allele frequencies of aeSNVs

Supplementary Data 24. Germline mutations in RCC

Supplementary Data 25. Primers of ATAC-seq
