# Supplementary material for: Genomic and epigenomic integrative subtypes of renal cell carcinoma in a Japanese cohort
Source: Nat Commun. 2023 Dec 16;14:8383. doi: 10.1038/s41467-023-44159-1 (PMC10725467; doi:10.1038/s41467-023-44159-1)
Supplement: Supplementary file 5 — Reporting Summary [file 41467_2023_44159_MOESM5_ESM.pdf]

nature portfolio

Corresponding author(s): Tatsuhiro Shibata

Last updated by author(s): 14th November, 2023

Reporting Summary

Nature Portfolio wishes to improve the reproducibility of the work that we publish. This form provides structure for consistency and transparency in reporting. For further information on Nature Portfolio policies, see our Editorial Policies and the Editorial Policy Checklist.

Statistics

For all statistical analyses, confirm that the following items are present in the figure legend, table legend, main text, or Methods section.

n/a	Confirmed	
☐	☒	The exact sample size (*n*) for each experimental group/condition, given as a discrete number and unit of measurement
☒	☐	A statement on whether measurements were taken from distinct samples or whether the same sample was measured repeatedly
☐	☒	The statistical test(s) used AND whether they are one- or two-sided<br>*Only common tests should be described solely by name; describe more complex techniques in the Methods section.*
☒	☐	A description of all covariates tested
☒	☐	A description of any assumptions or corrections, such as tests of normality and adjustment for multiple comparisons
☐	☒	A full description of the statistical parameters including central tendency (e.g. means) or other basic estimates (e.g. regression coefficient) AND variation (e.g. standard deviation) or associated estimates of uncertainty (e.g. confidence intervals)
☐	☒	For null hypothesis testing, the test statistic (e.g. *F*, *t*, *r*) with confidence intervals, effect sizes, degrees of freedom and *P* value noted<br>*Give P values as exact values whenever suitable.*
☒	☐	For Bayesian analysis, information on the choice of priors and Markov chain Monte Carlo settings
☒	☐	For hierarchical and complex designs, identification of the appropriate level for tests and full reporting of outcomes
☒	☐	Estimates of effect sizes (e.g. Cohen's *d*, Pearson's *r*), indicating how they were calculated

Our web collection on statistics for biologists contains articles on many of the points above.

Software and code

Policy information about availability of computer code

Data collection	No commercial software was used for data collection. All of the software used is reported in the paper.
Data analysis	Open sources used to analyze the data in this study are the following.<br>BWA-MEM (v.0.7.8): Mapping short-read sequences against a large reference genome, such as the human genome.<br>Bowtie (v.0.12.7): Mapping short-read sequences against a large reference genome, such as the human genome.<br>SAMtools (v.1.9): Remove PCR duplications and generate pileup files from mapped BAM files.<br>FitMS web tool (https://signal.mutationalsignatures.com/analyse2): a tool of analysing WGS tumour sample variants for the single base substitution signatures.<br>FACETS (v.0.6.0): Allele-specific copy number and clonal heterogeneity analysis tool for high-throughput DNA sequencing.<br>BLASTN (v.2.2.26): Search nucleotide databases using a nucleotide query.<br>GSEA (v.4.1.0): Computational method that determines whether an a priori defined set of genes shows statistically significant.<br>ChrAccR(v.09.11)): R package that provides tools for the comprehensive analysis chromatin accessibility data.<br>clusterProfiler (v.4.6.0): A universal enrichment tool for interpreting omics data.<br>Rsubread (v.2.4.3): Alignment and quantification of RNA sequencing reads.<br>DESeq2 (v.1.31.14): Estimate variance-mean dependence in count data from high-throughput sequencing assays.<br>matrixStats (v.0.60.1): functions operating on rows and columns of matrices<br>ChIPseeker (v.1.26.2): ChIP peak Annotation, Comparison, and Visualization tool.<br>HOMER (v.4.11): A novel motif discovery algorithm.<br>MEME Suite (v.5.0.5): Motif-based sequence analysis tools.<br>Macs2 (v.2.1.4): Tool for identifying transcription factor binding sites is named Model-based Analysis of ChIP-seq and ATAC-seq.<br>TOBIAS (v.0.12.10): Transcription factor occupancy prediction by investigation of ATAC-seq signal.<br>Bismark (v.0.20.0): A program to map bisulfite treated sequencing reads to a genome of interest and perform methylation calls.

March 2021

MethylSeekR (v.1.22.0): A tool to discovery regulatory regions.
CIBERSORTx web tool (https://cibersortx.stanford.edu): A program to assess tumour-infiltrating immune cells from RNA-seq data.

For manuscripts utilizing custom algorithms or software that are central to the research but not yet described in published literature, software must be made available to editors and reviewers. We strongly encourage code deposition in a community repository (e.g. GitHub). See the Nature Portfolio guidelines for submitting code & software for further information.

Data

Policy information about availability of data

All manuscripts must include a data availability statement. This statement should provide the following information, where applicable:

- Accession codes, unique identifiers, or web links for publicly available datasets
- A description of any restrictions on data availability
- For clinical datasets or third party data, please ensure that the statement adheres to our policy

Japanese 287 RCC cases' raw sequencing data (WGS 128, RNA-seq 287, ATAC-seq 72, Methyl-seq 64) generated in this study have been deposited in the European Genome-phenome Archive (EGA) under accession EGAS00001006919 [https://ega-archive.org/search/EGAS00001006919]. These data are available under restricted access because they are personally identifiable data defined by Japan's Personal Information Protection Law. Requests for academic purposes only will be processed by the ICGC Data Access Compliance Office [https://docs.icgc-argo.org/docs/data-access/daco/applying] within ten business days. After access has been granted, the data is available for two years. TCGA data we used are deposited under dbGaP Study Accession phs000178 [https://www.ncbi.nlm.nih.gov/projects/gap/cgi-bin/study.cgi?study_id=phs000178.v11.p8]. PCAWG data are deposited in Synapse [https://www.synapse.org] under accession syn11726616. Human genome reference (GRCh37) was downloaded from the UCSC genome browser [https://hgdownload.soe.ucsc.edu/downloads.html#human]. Source data are provided with this article.

Field-specific reporting

Please select the one below that is the best fit for your research. If you are not sure, read the appropriate sections before making your selection.

☒ Life sciences ☐ Behavioural & social sciences ☐ Ecological, evolutionary & environmental sciences

For a reference copy of the document with all sections, see nature.com/documents/nr-reporting-summary-flat.pdf

Life sciences study design

All studies must disclose on these points even when the disclosure is negative.

Sample size	No sample size caluculation was performed because the present study is observational, not interventional.
Data exclusions	Detailes of the exclusion criteria are reported in the paper. Samples not passing quality-contorol have been excluded.
Replication	Not applicable for this observational study.
Randomization	Not applicable for this observational study.
Blinding	Not applicable for this observational study.

Reporting for specific materials, systems and methods

We require information from authors about some types of materials, experimental systems and methods used in many studies. Here, indicate whether each material, system or method listed is relevant to your study. If you are not sure if a list item applies to your research, read the appropriate section before selecting a response.

Materials & experimental systems

n/a	Involved in the study	
☐	☒	Antibodies
☒	☐	Eukaryotic cell lines
☒	☐	Palaeontology and archaeology
☒	☐	Animals and other organisms
☐	☒	Human research participants
☒	☐	Clinical data
☒	☐	Dual use research of concern

Methods

n/a	Involved in the study	
☒	☐	ChIP-seq
☒	☐	Flow cytometry
☒	☐	MRI-based neuroimaging

Antibodies

Antibodies used	PD-1 (catalog number ab234444, colon NAT105; Abcam, Cambridge, UK), mouse monoclonal antibody, a dilution of 1:100

Validation	The antibody was validated by immunohistochemistry with the human lymph follicle.

Human research participants

Policy information about studies involving human research participants

Population characteristics	Two hundred eighty-seven Japanese renal cell carcinoma cases were collected. The age at diagnosis of 287 patients ranged from 6 to late 80s, and the mean was 62. See Supplementary Table 1 for details.
Recruitment	Renal cell carcinoma patients in National Cancer Center (Tokyo, Japan) were recruited with consent. There is no potential self-selection or other biases in this study.
Ethics oversight	the Research Ethics Committee of the National Cancer Center (approval number G20-03)

Note that full information on the approval of the study protocol must also be provided in the manuscript.
